# Racialization, colonialism, and imperialism: a critical autoethnography on the intersection of forced displacement and race in a settler colonial context

**DOI:** 10.3389/fsoc.2023.1171008

**Published:** 2023-08-29

**Authors:** Jennifer Ma

**Affiliations:** School of Social Work, McMaster University, Hamilton, ON, Canada

**Keywords:** Vietnamese refugees, racialization, colonialism, imperialism, forced displacement, critical autoethnography

## Abstract

Migration has been identified as a priority area for policy responses by both the federal and provincial/territorial governments yet, much of our knowledge about migration is not premised on addressing current xenophobic and racist narratives about migrants. The purpose of this research is an interrogation of Canada's colonialism, imperialism, and racialization, which produce specific oppressive policies and practices that have impacted my family. This research is premised on the understanding that in the space between what is known about migration in Canada and what is not, a great deal of narrative and interpretive work is done that makes assumptions about migrants, specifically forcibly displaced people from the Global South. Through a critical autoethnography focused on my lived experiences as a descendant of forcibly displaced Chinese-Vietnamese people living in a settler colonial nation state, this study critiques to what extent these assumptions are founded, and to what extent they represent a socio-political climate in which migration is set out as particular problems requiring a legal and policing solution. In particular, my analysis centers anti-colonialism and anti-racism, shifting to resistance to systemic violence and liberation, while considering the discursive and on-the-ground effects of racist, colonial, and imperial policies and practice. Set against the backdrop of the rise of white nationalism, xenophobia, and racism across all levels of government and academia, and the general public, the results of this study produce a counter-narrative focused on the intersection of forced displacement and race in a settler colonial context, which is both timely and urgent.

## 1. Introduction

When I was growing up, I was disconnected from my family's history and their journey to the nation now known as Canada. I did not understand the circumstances that led to our displacement and how they continued to affect me. It was not really until my undergraduate studies that I learned that my family were refugees who fled following the war in Vietnam in the late 1970s. The silence that surrounded our lived experiences created a situation where I felt untethered from my ancestors and unsure of where I belonged. I now understand that this is because my family has struggled and continues to struggle with the effects of the war, trauma, and displacement. I still do not have a full picture of what happened. I get snippets here and there, and to this day I am still learning about these fractured memories and stories from my elders while filling in the gaps with research, literature, and conversations with other Vietnamese and Chinese-Vietnamese refugees.

The purpose of this paper is to counter assumptions made about the Vietnamese diaspora in Canada, specifically people who were refugees. These assumptions include: the construction of the grateful refugee, the model minority myth, and the perpetual foreigner stereotype, Through a critical autoethnography focused on my experiences as a descendant of forcibly displaced Chinese-Vietnamese people living in a settler colonial nation state, this study critiques to which degree these assumptions are founded, and to which degree they represent a socio-political climate in which migration is set out as a particular set of issues requiring a legal and policing solution. My analysis centers anti-colonialism and anti-racism, shifting to resistance to systemic violence and the support of collective liberation, while considering the discursive and on-the-ground effects of racist, colonial, and imperial policies and practice. I wrote this not with the intention of representing everyone who is a part of the Vietnamese diaspora as this is not possible due to the varying experiences amongst the community. Rather, I wrote this to share my story in hopes of keeping the conversation going. This paper is part of a Special Issue entitled, Bodies at the Borders: Analyzing the Objectification and Containment of Migrants at Border Crossing and edited by Ryan et al.

## 2. A bit of herstory

My ancestors are Teo Chew nang, from the coastal region of the Guangdong province in China. They migrated to Vietnam from China in the early 1900s, displaced by war and drought. Several generations of my family were born and raised in the southern part of Vietnam while the nation was colonized by France and then briefly by Japan. They experienced a slow process of decolonization led by Ho Chi Minh, the leader of the Communist nationalist movement the Viet Minh. In 1946, an anti-colonial war began with Communist nationalists. The United States (US) got involved and spent $2.5 billion supporting France (Menand, 2018). In 1954, France lost and negotiated a settlement, the Geneva Accords, that partitioned the country at the seventh parallel until 1956 when a democratic election would be held. All parties agreed except for the US who did not want to see communism spreading throughout Asia. An election was never held. As a result, my family survived a decades long civil war produced by Western nations, outsiders who decided to split Vietnam in half, creating a division that has lingering effects on the community to this day. North Vietnam was governed by Vietnamese communists and South Vietnam was backed by American aid and eventually American troops. Violations happened at either end with violence deliberately inflicted on civilians as assassinations and massacres were carried out while weapons of mass destruction were dropped across the nation.

The US planted Vietnamese people to control South Vietnam—“freely” elected corrupt officials to be switched out when the previous one failed. This method was facilitated by $1.5 billion in aid between 1955 and 1961 (Menand, 2018). Even by 1963, when peaceful coexistence was the policy of American and Soviet governments, the US began their strategy of military escalation. They were repeatedly warned about the recklessness of their involvement, yet they continued to choose war. In 1963, there were 16,000 American advisors in South Vietnam (Amadeo, 2022). Over the next decade, about 3,000,000 soldiers would land there (Flitton, 1999). By the time they left, the US had dropped 6.1 million tons of bombs, more than three times as many as the Allies dropped during the entirety of the Second World War (Miguel and Roland, 2005).

April 30 in 1975 is known as the fall of Saigon, Black April, or *tháng tu den*. It is a date that the global diaspora grieves yet it is celebrated in Vietnam to this day. Over the next 20 years, between 1 and 1.2 million people left Vietnam by boat. The number of people leaving peaked in 1978 and 1979—the years both my paternal and maternal families left. One of the roots of this mass exodus was the government in Hanoi targeting ethnically Chinese people and abolishing “bourgeois trade” in the south (Chan, 2006) resulting in dispossession, persecution, imprisonment, and massacres. My family has spoken about how the government conducted raids before they could do anything about it. Their small businesses were eventually shut down as a result. The government then changed the currency, devaluing it, and as a result, my family lost their financial resources overnight. During this time, my aunt recalled hearing a message on the radio that explicitly told people of Chinese ethnicity to leave the country.

Caught in the middle of tensions between Vietnam, Cambodia, and China due to Vietnam's dispute with the latter nations in 1978 and 1979, Vietnamese people of Chinese descent had two options: to leave Vietnam or submit themselves to re-education camps where they would be tortured, starved, enslaved, and murdered. They were targeted as French colonial support of Chinese people's participation in commerce over the Vietnamese population resulted in their control of commerce in South Vietnam. As such, restrictions on economic activity following reunification resulted in my family seeing no future for themselves in Vietnam. They were forced to sell their homes and belongings to survive, leaving their lives behind to get on a derelict boat in the middle of the night with their loved ones. The government profited from the exploitation through the cost of exit fees and documentation, which my family told me was up to the equivalent of $3,000 for adults and $1,500 for children. Of those who left during this period, 800,000 people arrived at the shores of Malaysia safely despite pirates, violence, and storms (Vu, 2007). This included my family members and our community. The rest, approximately 200,000 to 400,000 people died at sea during the weeks-long journey (United Nations High Commissioner for Refugees, 2000).

To prevent people from settling in Malaysia the government housed half of the people who arrived under severely crowded and inhumane conditions on small islands off the coast.

When my paternal family arrived at the shores of Malaysia in 1979, they were sent to a refugee camp on Pulau Bidong, an isolated island off the east coast of Malaysia. The island was meant to house 4,500 refugees, but by a year after the temporary camp was set up the number of refugees had risen to 40,000 people (Thompson, 2010). When my father has spoken about fleeing Vietnam, he is clear that the refugee camp was worse than the boat journey itself. He recalls the dehumanization people experienced, witnessing violence, having to search for debris from boats to build shelter, the unsanitary conditions of the camp, and the lack of clean water. However, while there was devastation and violence at the refugee camp, there was also reciprocal support and collective care among the community. My family have told me stories about banding together to search for food and firewood, setting up a resource distribution system, and sharing knowledge about the resettlement process.

Eventually, Malaysia began to deny entry to boat people and would even push boats that arrived back out to sea (United Nations High Commissioner for Refugees, 2000). The Association of Southeast Asian nations warned that they would not be accepting any more refugees arriving by boat (Kumin, 2008). In response, The United Nations invited 65 governments to a conference in Geneva (Kumin, 2008). As a result of the 1979 Geneva Conference, Vietnam agreed to stop illegal departures and provide a system for people leaving the nation. Asian countries, including Malaysia, Indonesia, and Thailand agreed to stop turning away boats. Lastly, in exchange for providing temporary asylum, Western nations agreed that refugees would be resettled in the Global North, including the settler colonial nations, such US, Canada, and Australia, and European nations including France. In the end, between 1955 and 2002 the war and political violence led to a democide which resulted in the loss of a total of 3,800,000 lives or approximately 1 of 10 people of the overall population of Vietnam (Rummel, 1997; Obermeyer et al., 2008). Among these people, about 1,250,000 or one-third were murdered by foreign soldiers, including those from France and the US, and by the governments of North and South Vietnam in the struggle for control of the nation state (Rummel, 1997).

Once they were privately sponsored to Canada, my family and community were expected to assimilate and assume the role of grateful refugees, rejecting communism while supporting Western democracies (Ngo, 2016a). Since then, our lives have been shaped by the Canadian state project to “settle, adapt, and integrate” and contribute to the capitalist system, while being grateful for being rescued by the state—even though Canada participated in and was complicit in the war in Vietnam (Price, 2011; Nguyen, 2013; Ngo, 2016a). To this end the literature problematically constructs Vietnamese Canadians as productive refugees, describing us as a model minority in education, adaptation, and participation in capitalism (Ngo, 2016b; Hou, 2021; Nguyen, 2021). In doing so we are essentialized and perceived as acclimatized, assimilable, and economically successful (Espiritu, 2006, 2014; Nguyen, 2012, 2013, 2018; Ngo, 2016b). We are expected to overcome our trauma and struggles to gain financial independence, so we are no longer a burden to the system. We are expected to be hardworking, resourceful, and successful with minimal assistance from the state, while being committed to the state (Nguyen, 2013). Using us as a success story of refugee rescue and resettlement in the Canadian context, we are constructed as “exceptional,” erasing the significant struggles many of us continue to face because of structural and systemic barriers in Canada. As a result, our diverse struggles and needs are not seen by researchers, policy makers, and practitioners.

It is important to note that in the Canadian context, Vietnamese people have been subsumed in the Asian racial identity category and as such are subject to anti-Asian racism. Generally, Asians are stereotyped as being the model minority and at the same time perpetual foreigners. This dehumanizes us and sets us up as objects to be exploited for our labor, as exotified objects for consumption, or as a wedge to oppress other racialized people, including Black and Indigenous peoples. A critique of the term Asian is necessary here as it is a socially constructed group that reproduces essentialism. According to Lowe (1991), it is a monolith label and the monoracialism of Asians upholds racist systems that categorize Asians as a homogenous group. In this way our unique experiences are erased. However, it is important to reflect on being a part of a racial group that is targeted because they are perceived as belonging to the group. This offers an opportunity for working collectively to resist systemic violence toward people who identify and/or are seen as Asian.

The stereotype of Asian people as perpetual foreigners is based on orientalism (Said, 1978), the imagination of the Middle East and Asia as the land of the “other,” different, and exotic. This connects to imperialism and colonialism, whereby the American militarization of Asian land is glorified, and Asia is imagined as being saved by Western imperial militarism. Despite the reality that some migrants, particularly those from the Global South, have their own histories of colonialism and genocide, they often arrive in Canada with the illusion that fairness and equity are rooted in the values of the nation, when in fact, a history and legacy of colonialism and racism shows the opposite. Moreover, model minority theorists show how the construction of Asians as the desirable immigrant subject dismisses and delegitimizes the political claims of Indigenous peoples and non-conforming racialized others in the Global North (Park, 2011; Ku, 2012; Ngo, 2016a,b). Within the model minority discourse, Asian peoples' successes are attributed to their “culture” which comprises hard work, self-reliance, and a focus on educational attainment. Thus, Asian peoples' successes are linked to “cultural” factors, implying that other groups' issues are also linked to cultural factors rather than structural racism, sexism, homophobia, and classism (Ngo, 2016b). Pon (2000) discusses how the model minority discourses reinforces the liberal belief that Canada and its institutions are fair, accessible, and accommodating to people who work hard enough.

## 3. Contextualizing my critical autoethnography in response to colonialism, imperialism, and racialization

### 3.1. Colonialism

In the 1500s, European colonizers referred to Indigenous lands as feminized, racialized, inferior, and open for domination. From this orientalism (Said, 1978) grew, which led to imperialism and colonialism. A couple of historical examples are the atrocities committed against Vietnamese women during French colonization and then during the American war in Vietnam. It is important to note Vietnamese resistance to French colonization. For the US got involved once Vietnamese nationalists successfully overthrew French colonial rule. This is indicative of white supremacist colonial capitalism which is grounded in the multiple logics of slavery, genocide, and orientalism wars (Smith, 2006).

Historical and ongoing colonialism and racism must be acknowledged, as there is a prevalent ontology of forgetting this history in Canada (Razack et al., 2010). This ontology of forgetting is taken up by Vietnamese diaspora as there are a lack of opportunities for learning about the experiences of and building relationships with Indigenous peoples. As a result, the genocide of First Nations people is forgotten, as is the history of white supremacy, racism, and Western imperialism, which were fundamental to the construction and development of North America (Lowe, 1996). This ontology of forgetting is pervasive in the Canadian imagination about the Vietnamese community whereby anti-Asian racism and white supremacy are rarely mentioned. Indigenous peoples and Vietnamese refugees, among other racialized refugees, share distinct histories of colonialism, dispossession, racism, and exclusion. Both communities have been affected by the codification of racism through education and Canadian legislation and policies, such as citizenship and immigration laws, which supported the development of a privileged White national population with rights and access to resources that non-White people were historically excluded from.

A debate exists over the notion of migrant settler colonialism in the Canadian context. Lawrence and Dua (2005) suggest that racialized immigrants are settlers as they are complicit in and benefit from the settler colonial project. On the other hand, Sharma and Wright (2008) responded by critiquing the conflation of migration and colonialism. This debate has led to scholarship navigating global imperialism and white supremacy, which connect immigrant and Indigenous peoples in geopolitical spaces while considering the possibilities for solidarity. What has come out of the literature is the anti-racist concept of immigrant settlerhood (Chatterjee, 2019), which considers Indigenous self-determination and accountability for immigrant settlement on Indigenous land. Tuck and Yang (2012) followed up by discussing the irreconcilability of immigrant and Indigenous justice movements. Chatterjee (2019) argues that the separation of immigrant labor exploitation and Indigenous land dispossession conceals the settler political economy. She suggests that doing so ignores the realities of historic and ongoing capitalist, colonial exploitation of land and labor, racialized precarity, and the legitimization of White settler colonialism. Chatterjee (2019) states that settler colonialism, Indigenous dispossession, and immigrant settlement are intertwined social, historical, economic, and political practices.

Indigenous peoples and racialized migrants, including Vietnamese refugees, are connected via the capitalist colonial project of dispossession and precarious resettlement, which occur simultaneously (Chatterjee, 2019). While they experience different yet co-exiting colonial projects in distinct contexts, they share experiences of dehumanization, marginalization, deliberate impoverishment, and experiences of racism. Colonial assumptions about Indigenous peoples and racialized migrants have shaped how they have been treated through State policies and practices. These discourses include both groups being savages, deficient, and inferior compared to their White, settler counterparts. This has led to assimilative policies and practices to bring Indigenous peoples and racialized migrants closer to the dominant and normative culture. As such, knowledge, teachings, and skills brought to settler colonial nations are not recognized, valued, nor validated. A result of this is that racialized migrant communities are relegated to the lowest rungs of the labor market (Das Gupta et al., 2014). In addition to exploiting the labor of racialized migrants this also supports the dispossession of Indigenous claims to land through the management of immigrant populations by choosing who is a part of the nation state and labor market. According to Tuck and Yang (2012) this creates pathways to neo-colonialism for the possession of resources without unsettling the white settler domination of the nation state.

Following the Second World War and the 1947 *Canadian Citizenship Act*, the federal government managed immigration and citizenship under the Department of Indian Affairs, resulting in the establishment of the Department of Citizenship and Immigration (DCI). Up until 1966, when Indian Affairs was moved to a new ministry, its activities mirrored the citizenship policies created for migrants, relegating First Nations peoples as migrants on their own land (Bohaker and Iacovetta, 2009). Bohaker and Iacovetta (2009) posit that the DCI was part of a deliberate policy to control two groups perceived as threatening to the federal government, foreigners to be absorbed into White Canadian society. The initiatives aimed at First Nations peoples were less respectful of cultural traditions and political autonomy in comparison to those targeted toward European migrants. They were similar to the policy of assimilation that predated Confederation. In their comparison of the initiatives, “… the racial, gender, and class dimensions of a misplaced experiment to create a ‘one-size-fits-all' Canadian citizenship that, for all its talk of respect, tolerance, and common Canadian values, belonged to an ongoing project of white-settler nation building” (Bohaker and Iacovetta, 2009, p. 430).

In relation to Vietnamese refugees, while they seek citizenship and the promise of safety and security, Indigenous peoples have had settler citizenship imposed on them despite Indigenous sovereignty and their connection to the land, which is both natural and spiritual. In this way, the nation state socially organizes the everyday lives of the Vietnamese diaspora. Specifically, through the management of immigration and colonization the nation state controls and organizes people and activities at political, administrative, and institutional levels (Ng, 2006). As such, the bodies of racialized migrants are regulated and controlled by politicians, legislation, the Canadian Border Services Agency, and institutions such as the police, the criminal justice system, immigration, and the health care system (Ng, 2006). Generally, Indigenous peoples are not included or consulted in these processes. Specific to Vietnamese refugees, NGO resettlement workers played a role in both the micro and macro levels of the migration process by observing, helping, or hindering the ways in which refugees negotiated the selection process at the refugee camps (Vu and Satzewich, 2016). They held the power to decide who would be sponsored by which country.

A question I continue to grapple with is how is the land engaged with when people arrive in Canada—moving from one colonial nation state to another? How do we take into consideration the reality that some people who are seen as refugees are also experiencing their own process of de-indigenization? According to Adese and Phung (2021), “de-indigenization … refers to processes and programs geared toward irrevocably separating Indigenous peoples from their languages, cultures, familial relationships, ways of knowing, and lands” (p. 119). While there is the aspect of location and physical characteristics of the environment, there are also social or cultural meanings of place. How have Vietnamese people understood settler colonialism and how does it affect the ways they participate in society? Despite living in Canada and accepting it as their home while building a future for their children and grandchildren, Vietnamese people are deeply attached to the homeland and culture.

### 3.2. Imperialism

Settler colonialism is connected to imperialism through its enterprise of domination and exploitation both historically and contemporarily. Critical refugee studies have focused on the interrogation of imperialism and use queer and feminist critiques to identify, deconstruct, and analyze the violence that underlies the term “refugee” (Espiritu, 2006, 2014). This theoretical framework challenges discourses that implicitly or explicitly justify racial and gender hierarchies and US militarism in Vietnam and beyond. The extant literature focuses on how refugee nationalism, memory, assimilation, and identity are inextricably linked to war (Espiritu, 2006, 2014; Sahara, 2012) and to the institutions, governmental and non-governmental, that have affected refugees' resettlement experiences.

Specific to the Vietnamese community, Ngo (2016a) writes about how frameworks of meaning-making are deeply rooted in the events and effects of the Cold War. She discusses how the identities we occupy continue to be grounded in our experiences of being participants, victims, and witnesses to the civil war in Vietnam as part of the larger international Cold War conflicts (Ngo, 2016a). Cold War epistemology is an area of scholarship primarily by Asian scholars who interrogate the enduring knowledge production of Self and Other in Asians and Americans (Chen, 2010; Kim, 2010). According to Ngo (2016a) “it theorizes the ongoing impact of colonialism, imperialism, and the Cold War on the psyches and subject formation of Asian people and nations globally” (p. 68). Western imperialism in East and Southeast Asia has been carried out with the objective of “containing communism” as a physical and ideological threat to neoliberal democracy (Ngo, 2016a). Ngo (2016a) describes how this Cold War epistemology and related discourse exists beyond a historical event or series of events and has oozed into the psyche of the US/Western colonizer and Asian subjects.

Chen (2010) discusses the persistent impacts of Asian subjectivity and states that “the complex effects of the war, mediated through our bodies, have been inscribed into our national, family, and personal histories. In short, the Cold War is still alive within us” (p.118). This critical perspective provides a context for analyzing the social relations between the Vietnamese diaspora and the State that centers the Cold War. For it is the Cold War that brought waves of Vietnamese people to Canada to begin with and this political history continues to shape how the nation state and Vietnamese people relate to one another (Ngo, 2016a). According to Kim (2010), “Cold War compositions are at once a geopolitical structuring, an ideological writing, and a cultural imagining” (p.11).

As a result of this ideological positioning, the Vietnamese (as anti-communist) are considered “compositional subjects,” which can only be “visible” and “intelligible” in Canada through an understanding of the Cold War (Ngo, 2016a). This has led to conflict within the Vietnamese community as one specific Vietnamese subject is legitimized, while other identities are discursively excluded (Ngo, 2016b). Vietnamese American literature has called for more critical and nuanced analyses of Vietnamese refugee resettlement and political engagement in North America given the heterogenous socioeconomic and political positionings (Ngo, 2016b) and different pathways to citizenship (Bloemraad, 2006). The bodies of grateful refugees are scarred by the actions and consequences of the Cold War which continue to significantly affect the lives of people who have internalized the racist and imperialist conceptualizations of their bodies as subjugated persons who had to be rescued and given their humanity (Kim, 2010).

Espiritu (2006) examined the use of the Cold War and specifically the war in Vietnam as a meaning-making tool for the US, specifically the “we-win-even-when-we-lose” syndrome. She posits that American militarism was justified for the liberation of savage others in Vietnam, which is the same justification used in present conflicts such as the war in Iraq and Syria. As such, the War in Vietnam in the US imagination is simultaneously obscured and justified by the discourse of saving racially inferior others with the enforcement of so-called democracy and the “gift of freedom” (Nguyen, 2012).

Furthermore, Woan (2008) identifies the history of imperialism in Asia and its transgenerational effects as the roots of inequality for diasporic Asian women as we continue to be affected by colonial stereotypes. She introduces the theory of white sexual imperialism, in which rape and war have constructed the stereotype of hypersexualized Asian women. This is linked to Asian fetishization and white supremacy in North America beginning with the first wave of Chinese men migrating to work on the transcontinental railroad. Chinese women were excluded based on racist concerns of venereal disease (yellow peril) and being a threat to White civilization (Chan, 1991). So, while Asian women are perceived as deviant, foreign, and thus desirable, they are also a threat and this led to the exclusion of Chinese and other Asian women from migrating via the Chinese Exclusion Act, which was not repealed until the 1940s. In this context, we are dehumanized, objects for consumption and this is reinforced in contemporary Western media and films where we are often reduced to a sexual fetish or the sexual model minority.

Imperialism and colonialism in Asia have led to the commodification of our bodies and historical and contemporary violence against Asian women. From being harassed on the street to being raped and murdered we continue to be targeted in ways that repeat the atrocities committed during imperial wars. According to Woan (2008), “the Western world's desire for imperialistic domination over Asia relates to its desire for sexual domination over Asian women” (p. 301). White sexual imperialism provides an explanation for the inequality and violence Asian women across the globe face during contemporary times. This imperialist regime is the foundation for Asian women's experiences of sexual-racial oppression. If left unaddressed, violence against Asian women will continue to be perpetrated by White men and others while Asian women will continue to be seen through the lens of a hyper-sexualized stereotype. Woan (2008) further suggests that the intersection of imperialism and colonialism will provide a more comprehensive understanding of the oppression of Asian women.

### 3.3. Racialization

The façade of an innocent and neutral Canada has been chipped away at by the extant scholarship in critical multiculturalism. The inclusion of Cold War epistemology provides an opportunity for a deepened analysis of Vietnamese Canadians in relation to the state and nation building exercises. Critical theorists of Canadian multiculturalism have interrogated the strategies of Canada's nation-building as a white settler colonial project, including the construction and reproduction of the “desirable” vs. the “undesirable Other” (Bannerji, 2000; Thobani, 2007). Specifically, Indigenous peoples, racialized people, immigrants, and newcomers have been constructed as existing outside of the nation in multiple ways. In doing so, Canadian multiculturalism is employed as a governing tool whereby those who are not included in the nation are controlled and managed to serve the nation but to never fully belong within it (Ngo, 2016b). It is no coincidence that the federal government's implementation of multiculturalism as an official state ideology in 1972 happened at the same time as the Vietnam War and the mass exodus of Southeast Asian people. It was during this time that Canada shifted from being an overtly racist nation state to a pluralistic one (Beiser, 1999), supporting the constructed narrative of Canada as a tolerant and fair nation.

Similar to policies such as the *Indian Act*, racist immigration policies supported the maintenance of a privileged White national population with rights and access to resources, which non-White people were excluded from (Thobani, 2007; Maiter, 2009). From Confederation up to the 1970s, immigration laws limited admission to White people (Thobani, 2007). Non-White groups were considered “intruders” whose “inherent deviant tendencies” threatened the existence of the nation (Thobani, 2007, p. 75). Even though the discourse of legislation changed over time, the purpose of these policies was government control over the population. Racism was codified in immigration policies, including the 1885 *Chinese Immigration Act*. The Act included a head tax, which increased by 10 times over 3 years, which the federal government profited from while stopping the movement of Chinese people. Amendments to the *Immigration Act* were made, adding new reasons for denying entry and deportation such psychopathic inferiority and illiteracy to hide the blatant racism of the policies.

Racialization is understood as the circumstances by which certain social characteristics and behaviors come to be identified with race. The construction of the Vietnamese community as exceptional refugees occurred in the 1980s to support Canada's nation building project by maintaining their international reputation as a leader in humanitarian rescue and refuge (Ngo, 2016b). The humanitarian effort to resettle people forcibly displaced during the Vietnam War is an exceptional case as no other group of refugees has since been resettled in such large numbers. Because of this government actors, politicians, journalists, and legal and health professionals were interested in their lived experiences. This resulted in cooperation across all levels of government and the voluntary/private sector and policy or service reports that informed supporting newly resettled Vietnamese people (Knowles, 1997). Most of the extant literature is primarily focused on acculturation into Western society. Historically, these discourses reproduced the notion of Asian people as foreigners on a pathway to assimilation (Yu, 2002). Recent research has interrogated narratives of freedom and the model minority myth, while contesting the cultural biases of assimilationist frameworks while centering experiences of economic exploitation, institutional and structural racial discrimination, and other systems of oppression (Nguyen-Vo, 2005; Nguyen, 2021; Peche et al., 2022).

Another layer is Canada's colonial myth that has reinforced and continues to reinforce the construction and narrative of Canada as a vast, unoccupied nation founded by French and British colonial settlers. This myth serves to silence and erase Indigenous claims to sovereignty and the land, as well as the history of Black indentured people, Chinese laborers, and racialized settlers. Ngo (2016b) discusses the ways in which these communities have contributed enormously to the nation state in material, cultural, social, and political ways, yet their histories are minimized as their descendants continue to experience racism and xenophobia as outsiders to the nation. Specific to the Vietnamese community, the discourse of them being exceptional refugees is an integral part of the reproduction of multiculturalism as it holds them up as legitimate refugees compared to other racialized groups who are constructed as illegitimate and therefore undeserving refugees (Ngo, 2016b; Nguyen, 2021).

## 4. Who am I in relation to this work?

I identify as a Southeast Asian queer woman living in the settler colonial nation state known as Canada. I was born and raised in Tkaronto/Toronto and currently reside there. My immediate and extended family are from Vietnam. Our ethnic and ancestral lineage is Teo Chew. Although I have visited Vietnam, I have never lived there. I was the first born in Canada, 6 years after my family arrived. I was raised by my parents, aunts, uncles, and grandparents. Throughout my life I have struggled with my identity and culture. My experiences of my racial identity were focused on how I looked as well as my ethnic identity. I have been told to go back to where I come from too many times to count. I did not speak English when I entered the colonial education system so children teased me, and teachers continually underestimated me. I was one of the only few Asian girls at school and I felt out of place and different. I was excluded and bullied for being Asian and remember hating being Chinese and Vietnamese. This made me angry. I felt ugly with the implicit meaning that I did not fit within dominant, Eurocentric beauty standards, and then when I grew up was objectified, exotified, and sexualized as an Asian woman. My parents expected me to succeed academically, and I felt a lot of pressure to perform. Professionally, my accomplishments and successes have been devalued and attributed to my racial identity based on the model minority myth.

What my family and community survived—from the war, to planning their escape, to time spent in a refugee camp, to resettlement—demonstrates that telling the truth about our lived experiences may result in death. As a child, I learned to remain silent and invisible as a means of survival. Sadly, this meant I engaged in assimilative practices, such as performing whiteness. I began to lose my Chinese and Vietnamese cultures as I grew up. I have spent years healing from internalized racism and issues surrounding belonging, and it is an ongoing process. I started asking my family questions about our history, about our identities and our cultures. As of late I have been spending time reconnecting to my culture and have been working as a social worker in the community serving Vietnamese people seeking mental health support. It has been such a meaningful experience to come full circle and to be able to hear the stories of folks who are willing to share the things we do not talk about.

In the Canadian context, I do not fit into the Indigenous/settler binary. I did not choose to be born here. I was only born here because my parents met for the first time in Chinatown in downtown Toronto one fateful day 2 years after their separate arrivals. It is difficult for me to see myself as a settler because my family were forcibly displaced due to colonialism and imperialism. I recognize the parallel yet distinct processes of colonial violence and struggle for decolonization that my family faced and that which Indigenous peoples across Turtle Island face. I understand us to have more shared experiences, such as colonialism and imperialism, rather than differences. At the same time, I am conscious of how this represents a move to innocence (Tuck and Yang, 2012). These are complex issues I am still navigating and the only thing I am sure of is the sense of placelessness that I feel and embody. Where do I belong? Neither here nor there.

## 5. Methodology

Critical race theory (CRT; Delgado and Stefancic, 2012) traces racism through the legacy of slavery, the Civil Rights movement, and contemporary events, including the dynamic ways racism and white supremacy are perpetuated through institutions and policies. CRT focuses on the construction of race and racism across dominant cultural modes of expression, including law and policies, research and education, language, literature and film, and art and media. Specifically, it describes how targets of systemic racism are affected by cultural perceptions of race and how we can represent ourselves to systems through counterstories. Furthermore, there is an emphasis on social activism and transforming everyday notions of race, racism, and power through resistance and an intersectional approach. I decided to engage in a critical autoethnography to center my voice and counterstory to mainstream narratives which erase the lingering effects of imperialism, colonialism, and racism among the Vietnamese diaspora.

The extant literature positions autoethnography written by people who have been/are colonized as “[troubling] the concepts and categories we breathe in, think through and live in” (Dutta, 2018, p. 95). The analysis is rooted in lived experiences and thus amplifies the silenced voices of those of us who are underrepresented and oppressed (Chawla and Atay, 2018, p. 3). In this way, autoethnography not only highlights the realities of colonization but also “offers a critically reflexive tool for decolonizing” (Dutta, 2018, p. 96). Grounded in the work of Frantz Fanon, Chawla and Atay (2018) explain how autoethnography can be decolonizing for both the colonized and colonizer. Through our narratives those of us who identify as colonized speak back to colonizing systems of power while speaking to colonizers or settler colonizers. This provides an opportunity for colonizers to engage in a deep process of self-reflection, in which the negative impact of colonization is recognized alongside the unearned advantages they have (Chawla and Atay, 2018, p. 5–6).

Through a critical autoethnography, which, “…describes or analyzes personal experiences to better understand a cultural event,” (Croucher and Cronn-Mills, 2015, p. 137) I describe and analyze my lived experience as a descendant of forcibly displaced Vietnamese people of Chinese ancestry living in a settler colonial nation state. I selected this critical qualitative method, as my experience was affected by imperialism, global colonialism, and systemic racism. I focused on an autoethnography in response to the lack of visibility of second-generation Chinese-Vietnamese experiences and the silence which shrouds the Vietnamese community, which has been isolating. In addition, in response to epistemic injustices experienced by my community I am centering my voice in my research. My hope is that sharing my experience will be powerful and meaningful for those who are part of the Vietnamese diaspora.

Critical theory is a theoretical paradigm rooted in the study of power. Specifically, critical theory is often used as a lens to examine the exertion of power, as well as the symbiotic relationship between oppression and privilege. The purpose of this is to shine a light on power differentials and the maintenance of the status quo on a cultural level. Critical theory is applied to critique reality, identify the failings, relating them back to the concept of power, and then to discuss the ways in which power is misused. It is a theory that has been used to discuss concepts of social and cultural identity such as race, gender, and class (Dimock and Cole, 2016). As such, this critical autoethnography was guided by the following research questions:

- To what extent do assumptions about Vietnamese refugees represent a socio-political climate in which migration is set out as particular problems requiring a legal and policing solution?
- How have we and how can we resist systemic violence while promoting collective liberation, considering the discursive and on-the-ground effects of racist, colonial, and imperial policies and practice?

## 6. The critical autoethnography

### 6.1. Private sponsorship and the surveillance of Vietnamese refugees

The 1976 Immigration Act allowed for the private sponsorship of refugees to be facilitated following the end of the war in Vietnam. The policy allowed for charities, non-profit organizations, or a group of five individual citizens to sponsor a refugee family by providing them with a place to stay, supporting them with securing employment, or enrolling them in academic studies (Knowles, 1997). In the late 1970s and early 1980s, about 7,000 private sponsor groups supported the resettlement of Vietnamese refugees in Canada. Between 1979 and 1980, during the second wave of migration from Vietnam, Canada resettled over 60,000 refugees from Indochina, with 57% of refugees being privately sponsored (Van Haren, 2021). People from this group, like my family, mostly fled the country aboard overcrowded and dilapidated boats, and as such all Vietnamese refugees were referred to as “boat people.” Ethnic Chinese people made up 70% of the boat people (Koh, 2016).

Since 1994, the private sponsorship of refugees comprises 25–40% of Canada's annual total resettlement rates (Canadian Council for Refugees, 2016), demonstrating the Canadian public's proactive participation in private refugee resettlement. Each province or territory was provided with a relocation quota based on their representation of the total population of Canada (Hou, 2021). Those who arrived in the second and third wave were farmers, fishermen, merchants or former military members from small provinces and rural areas. They arrived at a severe economic disadvantage in terms of English proficiency and marketable job skills. Many people experienced physical violence and psychological trauma from their journey by boat and their time spent in refugee camps, physical and psychological distress from re-education camps, and the pain of losing or being separated from loved ones (Lee, 2015).

When my father has spoken about the private sponsorship experience, he identifies some issues with the process, including cultural differences, language, education, a lack of job skills, and resettling in a different climate than Vietnam. He shared that the government matched funds that people were contributing meaning the government would sponsor one refugee for each one sponsored privately. What ends up happening is that private sponsorship creates tension between the government and sponsors over selection control and numbers, which are divergent. While sponsors support people above and beyond the government's commitments, sponsors have faced administrative and regulatory changes that result in them having to do more with less, and the knowledge that overall resettlement will reduce if they are not able to support more people (Labman, 2016). In this way, the government can place the blame on sponsors for not filling the gap while constructing a narrative that resettlement is not important nor supported by Canadian citizens (Labman, 2016).

My family have shared that private sponsors could take referrals for people they wanted to sponsor. This would usually include family members of people who were originally sponsored so that they could reunite with loved ones. Unfortunately, this stopped in 2002 when the government canceled the Assisted Relative Class of sponsorship to control who could become a citizen of Canada. My father worked as an interpreter when he arrived in Canada, and he noticed that the government had specific categories for choosing whom to sponsor which were quite strict. People had to be a certain age (employability) and they wanted to limit larger family sizes and married people. There was a preference of single men or women so they would secure employment as the government did not want to support a whole family. The government or private sponsors provided financial support for 1 year or until people became employed. However, this was a temporary fix to larger systemic issues.

My family wanted to gain employment quickly so they would not be dependent on their sponsors. We crammed three generations into one house for the first couple of decades since this is what we were used to in Vietnam, and we could not afford to live separately. The government would send agents to check in on people once a week, while my family's sponsor checked in with them regularly over the first few months. I struggle with this as I see this as surveillance however, my family has spoken about how connections were created between refugees and their sponsors. They shared their experiences of going to church with their sponsors, cottages, and family parties. In this way, private sponsorship transforms everyday interactions with refugees affecting the ways in which citizens understand themselves in relation to refugees. On the other hand, if people were sponsored by the government, they checked in, paying close attention to make sure their basic needs were met. Because Vietnamese refugees were considered stateless at the time, they could not be deported, but now people who emigrate could be deported or stripped of their citizenship.

My family were expected to secure employment and become financially independent. They took on low paying jobs and some members decided to return to school. From a young age, my parents encouraged my sister and I to go to school to advance our careers. My father and I have discussed the eternally good, grateful, and deserving refugee trope (Espiritu, 2006, 2014; Moulin, 2012; Nguyen, 2012), which is particularly prominent among literature and media about Vietnamese refugees in the Canadian context (Nguyen, 2013, 2018; Ngo, 2016b). We are constructed as grateful for being rescued from political persecution and being given an opportunity to rebuild and resettle, yet many of us resent the US government for abandoning South Vietnam, which led them to become refugees in the first place. Among the community, many people distrust democratic institutions, which they blame for the demise of South Vietnam. There is tension between my family and I as the survivors of the war are truly grateful for Canada and being able to resettle in this nation after almost losing their lives. On the other hand, I can only focus on the harms that the Canada had done to us, Indigenous peoples, and other racialized communities. My father talks about how there was no support system in Vietnam at that time, and that there was a better social support system in Canada. As such, they chose to go to Canada as they would have had to wait longer to get to the US where my extended family wanted to go because of the climate. Despite everything, my family would still choose Canada as the US is perceived as overtly racist and because they went and started a war in Vietnam and then left when they were defeated.

### 6.2. Erasure of our experiences

To this day, I still feel the distinct reality of being Chinese and Vietnamese while not quite being a part of either community. My family used to share snippets of stories of what it was like being ethnically Chinese in Vietnam. They were discriminated against, restricted to living in certain areas, and forced to speak Vietnamese. People of Chinese descent were killed for speaking up. When they decided they could no longer live in Vietnam my uncle was the first to leave as they could only secure one spot due to circumstances beyond their control. I grew up feeling resentful of my family for not sharing more about our journey sooner, however I now understand this silence because it is too painful to recall those memories. Some families were wiped out, there is a mistrust of the government, and people resent Americans and their action in Vietnam. When pressed for more information there is a limit, and my family often talks about the need to move on and forget about it. In the past, they did not want family members to worry since they have already been through so much. This has carried on to contemporary times, so we tend to keep our struggles to ourselves. This silence and the erasure of our experiences has been hard for me to navigate. While I understand why, it pains me that so much is left unsaid and therefore assumptions are made about each other opening the scars of the wounds from the war and our life in Canada.

In our community, there is the need to show face by demonstrating social mobility, success, happiness, and independence from the State, hiding the realities of poverty, violence, grief, and subjugation from external forces. As a result of this, I have struggled with intergenerational trauma and mental health and well-being because of the pressure to be successful academically and professionally and the barriers I have faced along the way. I feel an immense pressure to do all the things my parents did not have the opportunity to do. I am aware that succumbing to this pressure reproduces the stereotypes of refugees as hard-working people who can pull themselves up by the bootstraps and become responsible, wage-earning citizens. I do not want to feed into the idea of Canada as a successful multicultural state with fair immigration policies. When we participate in this system, we become citizens of the nation state as we move closer in proximity to whiteness—the cultural, legal, and aesthetic norms of the settler colonial nation. Ultimately, these frameworks position refugees as people who are rejected from and desire admittance to the property owning, heteropatriarchal family of Canada's liberal, multicultural society (Nguyen, 2021). While we may put forth images of success and happiness this erases our vulnerability to and realities of racism and systemic violence while reproducing a colonial, nationalist script. This script and the idea of Canada is a place that is free and fair for refugees conceals the ways in which racialized people from the Global South are excluded and regularly blocked from obtaining a visa to get to Canada. Despite this, many racialized migrants have resisted the categories imposed on them by the white supremacist, settler colonial nation state.

### 6.3. Resisting systemic violence

While the refugee camps were violent and despairing places where people were up against policies and processes beyond their control Vietnamese refugees were not completely powerless, hopeless, or passive. Beyond a survival strategy, Vietnamese refugees resisted against the settler colonial state which is organized to exclude and exploit migrants. While my family and community could not control the selection criteria of settler colonial nation states and were subject to racist policies and decisions, they were able to push back by using their knowledge and connections to cross borders and achieve their desired settlement outcomes. My family recalls speaking to others at the refugee camps to gain information which would then be used to construct stories and identities that would be desirable to Canada. This included changing their birth year, relationships to one another on paper, and embellishing family histories to fit into the ideal that the state was searching for.

Ngo (2016b) writes about challenging the claims of North American rescue and liberation and the humanitarian rhetoric of North American government actors. When my mother arrived in Montreal, she was told to throw her old clothes away and she was hosed down with disinfectant and then given new clothes as if doing so would change anything about her that was seen as undesirable. While nationalist discourses use refugee journeys to demonstrate the West's benevolence and the success of liberal humanist frameworks of justice and freedom, there are stories that do not allow us to celebrate the exaltation of the refugee figure into a welcoming, multicultural city. Instead, these stories show a multiculturalism that does not affirm the lives of postcolonial immigrants and racialized people as it claims. My family recalls how they realized they were racialized people or other when they were out and about and experienced racial discrimination—for example, they would get rude stares, people shouted profanities at them as they went to work, and neighbors would damage their belongings. My father speaks about how White people were skeptical of foreigners in a small town, in which there were mostly White people living. He shared that language and cultural barriers prevented them from making friends with White locals, but they befriended other Asians in the community, such as a Pakistani family, due to cultural similarities. Eventually, my family decided to move to Toronto since there was a Chinatown where they experienced more subtle forms of racism in Toronto as well as more diversity.

A big source of tension between my family and me has been my need to name systemic violence and my knowledge of Canada as a settler colonial nation. While my family understands both to be true, they often expect me to remain silent to protect whatever sense of safety and security they have been afforded because of living in Canada. I struggle with this because my family was born in a country that has resisted colonial and imperial violence for centuries. After they were granted asylum a year or so after the war's formal end, most of them entered a life of low-wage, manual labor, despite going to and being successful in postsecondary education. Although later in life, they were able to secure work that was not so hard on their bodies, there were generally very few opportunities for advancement, so they have lived relatively modest lives. Even though discourses of upward mobility after temporary struggle for refugees are prevalent, their lives were deeply structured by socio-economic and racial, as well as sexual, marginalization. Furthermore, racial capitalism, or the co-constitution of modern capitalism and processes of slavery, genocide, racial hierarchy, invasion, and settlement, prolongs the search for refuge, creating persistent forms of economic, racial, and physical displacement.

### 6.4. Engaging in collective liberation

There is a history of social activism in South Vietnam, which includes mutual aid societies, school clubs, professional societies, and philanthropic and civic organizations (Nguyen-Marshall, 2022). My family and community's experiences have demonstrated that refugees have resisted and organized politically to better their living conditions in refugee camps or in the nation states they were resettled in (Nguyen, 2013). My family tells the story of how as soon as they arrived at the camp, they sent letters to other camps to look for family members so they could be reunited and to demonstrate they had family connections to the government. As I mentioned earlier, they had conversations about where they would like to end up, adjusting their stories to meet the requirements of each nation's immigration policies. The government of Canada helped my family find my uncle who was the first to leave Vietnam and eventually thy met him in Kuala Lumpur at the Canadian transition camp.

Starting from the time they left Vietnam my family focused their time and energy on relationship building and engaged in activities that supported our family and community. There were growing Vietnamese communities which comprised networks of family and friends who supported one another in their journeys during and after leaving Vietnam and the refugee camps. My father told me that Vietnamese people who came before 1975 could not return so they volunteered to help. This included professionals who are highly educated and organized groups of people to give them guidance on their new life in Canada and information on how to navigate the city, from everything as basic as how to cross the street to how to secure employment. There was a Vietnamese Association, a non-profit, that provided ESL classes and supported people looking for work, job placement, skills training and taking them to the doctor. They also provided translation and interpretation services. My father went on to become a social worker to further support the Vietnamese community and disadvantaged populations in Canada.

What I have observed is that my family and community are healing alongside the Vietnamese diaspora through various forms of social networks, which are both formal and informal. We have provided support to each other in a multitude of ways on an ongoing basis. We have benefitted from existing community resources and networks whether navigating the resettlement process, supporting business operations, or in political participation and activism. We have engaged in extensive transnational social networks and civic engagement experiences that have shaped the cultural, economic, and politic life of the diaspora through the generations. We recreated a sense of home in a place where we were never meant to be. In terms of spirituality, my grandparents are prominent members of a Chinese Buddhist Society and have engaged in ancestor worship over the past 40 years. On the other hand, intra-community conflicts exist. Ngo (2019a) has discussed the ways in which Vietnamese refugees negotiate their sense of identity and community in a multicultural nation state and how this is affected by significant political forces including the Cold War legacy and settler colonial nation building defining who and what is a refugee.

## 7. Discussion

Multiculturalism as a discourse reproduces the colonial project of white settler societies through the inclusion and exclusion of racialized people. This is carried out through colonialism, imperialism, and racialization which is meant to divide and conquer Indigenous, Black, Asian, and other racialized people. As described earlier, in the late 1970s, 60,000 refugees from Southeast Asia entered Canada—this was the first time that the nation state had admitted a substantial number of racialized refugees. At the same time, this period is also signified by the height of postwar Canadian nationalism and attempts to project an image of liberal inclusion, which was followed by state-sanctioned multiculturalism in 1971. However, this national identity failed to address racial discrimination, including discrimination directed toward Asian immigrants from the mid-19th century up to the arrival of Southeast Asian refugees in the 1970s. While Canada's Cold War politics are informed by these unresolved traumas, the intersections between the experiences of Vietnamese refugees and the Cold War remain largely ignored by the nation state.

Ngo (2019b) examined the discourses of democracy and communism, which she describes as remains of the Cold War, and demonstrates how the concept of democracy is conflated with liberal freedom. Specifically, freedom of development and trade and the ways in which this freedom is interconnected to capitalism. In this way, capitalism is used to protect and increase the prosperity and security of the nation state, while ignoring the reality that Canada is a settler colonial nation which exists on stolen land. According to Ngo (2019b), in the mainstream, the Cold War binary is seen as democracy vs. communism, however in reality the binary is capitalism vs. communism. This discursive move works to render capitalism invisible to Canadian national identity. Ngo (2019b) adds that racism is also a key feature of this national identity. Racism continues to exist in Canada, yet the interconnected systems of oppression which uphold white supremacy on the foundation of colonialism and capitalism work to block attempts to name it and to redress it. As such, systemic racism in the forms of social, economic, and political exclusion continues unabated.

Recently, there was a move to enact the Journey to Freedom Day (Bill S-219), a national day commemorating the mass exodus of Vietnamese refugees and their acceptance in Canada (Public Works and Government Services Canada, 2015, p.1). The Canadian narrative contradicts Vietnam's National Day of Reunification, and Vietnamese Canadians expressed their reluctance to support this bill suggesting that the date be changed to July 27, 1979—the day Canada officially committed to admitting 50,000 refugees. Ngo (2016a) posits that this bill constructs Vietnamese people as a political subject in tension with those who identify otherwise and contributes to the erasure of the American war in Vietnam by reproducing discourses of freedom while concealing Canada's participation and complicity in the war. For example, Canada provided monitoring and surveillance at the demilitarized zone in Vietnam (Bothwell, 2000).

Canada's role in the Vietnam War went beyond surveillance, as Nguyen (2013) states: “it must be remembered that while Canada did not join the fighting effort, it acted as the chief arms supplier to the U.S., providing resources and materials that fuelled combat and drove the war economy” (p. 25). Canada produced traditional and chemical weapons that killed millions of Vietnamese civilians (Nguyen, 2018). The Journey to Freedom Day bill attempts to uphold Canada's “national mythologies” of innocence (Dua et al., 2005), which are employed to “erase the history of colonization, slavery, and discriminatory immigration legislation” (p. 1). A focus on freedom and discourses of Vietnamese exceptionality continues to have devastating effects on survivors, who continue to struggle with transgenerational trauma, chemical poisoning, the destruction of kinship ties, and the loss of spiritual and material resources (Ngo, 2016a).

I have continued to wrestle with identity, belonging, and community building as I attempt to come to peace with our past. I have struggled due to the erasure of the war in Vietnam and the deliberate forgetting of the conditions which resulted in the exodus and war-created refugees. This ontology of forgetting is part of the larger nation-building project (Thobani, 2007). The focus on our journey to freedom conceals the American atrocities of the war, from the carpet bombing of entire regions of Vietnam, the My Lai massacre, the napalm attacks, the broad unrestrained use of Agent Orange poison, and to the still active land mines which remain largely uncovered (Espiritu, 2014). In this context, Ngo (2016a) argues that social policy is used as a tool for nation building and as a method of knowledge production which upholds and reproduces subject positions, thus contributing to the context of conflict within groups.

My father and I have discussed the programs that the government developed and the reality that not all the programs were helpful as they were not tailored to the specific needs and vulnerabilities of refugees. For example, there needed to be a specific support system for language training geared toward certain segments of the population, e.g., tailoring services to people and their specific needs such as elders. English as a Second Language was a one stop shop which was not helpful because of varying degrees of English proficiency and upbringing (some people in Vietnam were well educated and could speak English). My father identified a lack of support for the elderly regarding housing and health care, which was a small population because elders chose to stay in Vietnam since they were near the end of their life and did not want to start all over again in a foreign nation. There was almost no support for post-traumatic stress disorders and people were left to heal on their own, which many did not have time to do as they had to focus on working to survive. In addition to this, there were barriers and challenges in accessing adequate healthcare services compared to people who were citizens. Overall, people were excluded from decision-making processes about issues that directly affected them. Instead of relying on private or non-profit agencies, the government should have supported community-led organizations to develop communication campaigns, the provision of essential services, conduct contact tracing, and to support the development of social norms.

### 7.1. Assumptions about Vietnamese refugees and surveillance by the state

Through an analysis of my lived experiences and those of my family and community, I have countered assumptions made about Vietnamese refugees in Canada, including: the construction of the grateful refugee, the model minority myth, and the perpetual foreigner stereotype. These assumptions work to maintain control of the Vietnamese diaspora by rendering experiences of systemic discrimination invisible while reproducing the idea that Vietnamese people are racialized others who do not belong to Canada but should be eternally grateful for refuge. Pfeifer (1999) discussed temporal themes in the mainstream media's portrayal of Vietnamese people focusing on media coverage in Toronto, Canada from 1979 to 1996. Initially, the coverage detailed the perilous journey of the boat people from fleeing Vietnam to the conditions of refugee camps. These stories worked to garner support for refugees and increased the number of private sponsorships. In response to public anxiety over potential negative societal impacts because of admitting large numbers of Vietnamese refugees, newspapers started to publish pieces focused on the economic contributions of previous waves of refugees. As a result, we were dehumanized and reduced to bodies for labor, particularly manual, low-income labor. At the same time, several mainstream publications raised concerns about the high expense and potential impact of the “Boat People” upon the job market, the low-income housing sector, public health, and the social service delivery system (Pfeifer, 1999).

In the 1980s, there was a predominant focus on assimilation issues and experiences relating to systemic discrimination among Vietnamese refugees, which included “culture shock,” language difficulties, mental health problems, unemployment, and youth gangs (Pfeifer, 1999). Pfeifer (1999) identified the persistent reference to people of Vietnamese descent as “refugees” and “boat people” as a common theme in the articles, which started during the 1980s and has continued in media coverage of stories involving the Vietnamese population to this very day. At what point do we stop being refugees and when will we belong or be considered Canadian? Mainstream discourses reproduce the assumptions of the grateful refugee, the model minority, and the perpetual foreigner by discussing individual achievements and our ability to overcome significant adversities.

During the 1990s mainstream discourses about the Vietnamese diaspora shifted to focus on criminal activity and this has continued in contemporary times. Pfeifer (1999) showed that publications mostly discussed Vietnamese involvement in crime while a smaller number focused on Vietnamese-community activists opposing the collection of race-based crime statistics in the early 1990s, protests concerning media portrayals and police mistreatment, the lack of funding for Vietnamese social service organizations, and experiences of prejudice, racism, and violence directed toward Vietnamese people by representatives of the criminal justice system, the labor market, and schools. There were several articles that focused on narratives of the model minority myth, sharing the story of Vietnamese people who achieved upward mobility and youth who excelled academically.

The mainstream media is a key institution, which promotes racialization processes through the reproduction of dominant discourses. Pfeifer (1999) demonstrates that frequent references to Vietnamese individuals accused of involvement in criminal activity appeared in mainstream Toronto newspapers. He identifies three elements in media coverage that have supported the formation and perpetuation of negative stereotypes and the racialization of the Vietnamese diaspora in public consciousness. These include identifying the race of racialized people in newspaper stories relating to criminal activity and deportation when it is not relevant, the assumption of cultural factors as explanations for criminal behavior among the Vietnamese community and the correlation made between incidents involving Vietnamese people and Toronto neighborhoods, specifically Chinatown, Parkdale, and Jane-Finch, and often negative public connotations.

Vietnamese Canadians have experienced prejudice, discrimination, and mistreatment in their interactions with representatives of the criminal justice system in the Toronto area, specifically law enforcement officers (Pfeifer, 1999). The police have harassed Vietnamese people, asking for their identity in various situations. This harassment also extends to mall security staff and false accusations of shoplifting. Language barriers exacerbate these issues, from being charged with crimes because of an inability to communicate effectively with law enforcement in English to lawyers not adequately explaining court processes and being pushed to plead guilty. These realities combined with mainstream discourses about the Vietnamese diaspora demonstrate that the assumptions and stereotypes of Vietnamese refugees represent a sociopolitical climate in which migration is constructed as an issue requiring a legal and policing solution to control Vietnamese bodies.

### 7.2. Challenging coloniality

Negative attitudes and behaviors toward immigrants are a result of democratic racism (Henry et al., 2006), which prevents the government from truly embracing differences or making substantial changes to the existing social, economic, and political order. It also prevents them from supporting policies and practices that might disrupt the status quo, as these policies are understood as in conflict with and a threat to liberal democracy (Guo, 2010). There is a need to dismantle discriminatory policies and barriers through an inclusive framework that values all human knowledge and experiences. An example is the interrogation of the points system and the Eurocentric recognition of Canadian equivalency using white supremacist measurement criteria which claims to be objective, but is not. These are issues I teach students about in my role as a post-secondary educator who teaches at a colonial institution. I engage my students in critical conversations about colonization and its connection to white supremacy, racism, and capitalism—linking local issues to global issues. We talk about the ways in which colonialism manifests in the educational system and knowledge production. As a social work educator, I teach students about the roots of social work as a colonial profession that was and continues to be complicit and to participate in the systemic oppression of Indigenous, Black, and other racialized communities such as through the child welfare system.

I recognize that decolonization is not a metaphor (Tuck and Yang, 2012), rather it is a social and political process that seeks to recover and re-establish marginalized cultural knowledge, practices, and identity (Smith, 1999). As such, I am unlearning and relearning as an ongoing, lifelong process. I am also engaged in reconnecting to ancestral worldviews, teachings, and practices and encourage students to do so as well. I teach with the goal of resisting epistemic injustices and epistemicide (Santos, 2014). I center knowledge that is culturally relevant to transnational migrants through the diversification of knowledge production and the recognition of plural systems of knowledge (Santos, 2014). Students spend ample time thinking about our complicity in systems of oppression, specifically our participation in a settler colonial nation state, moving toward respect, responsibility, and reciprocity. I speak out about anti-Asian racism, anti-Black racism, anti-Indigenous racism, etc. without comparing the different experiences of racism which reproduces racial hierarchies. I understand that while they are different, they are connected through a global white supremacist colonial capitalist project that displaces and dispossesses Indigenous peoples around the world through the theft of land and resource extraction. In the classroom we discuss land back movements and the consideration of Indigenous treaty rights and relations (Tuck and Yang, 2012).

It is of utmost importance to me to develop meaningful and critical relationships between Indigenous peoples and refugees to live alongside one another while developing an ethics of care and accountability to one another (Adese and Phung, 2021). Our claims to Canadian resources such as land, add to the further dispossession of Indigenous peoples. On the other hand, for colonial-capitalist forms of extraction, the perpetuation of a conflictual relationship between Indigenous and immigrant populations fuels the reproduction of the white settler domination. To critically challenge this, it is important to engage in contextual collaboration and solidarities based on the mutual recognition of marginalization and oppression by the same logic of colonial-capitalist extraction (Stanley et al., 2014; Chatterjee, 2018). These solidarities do not have to be complete, unconditional ones, instead they should be based on a responsibility to advance living a dignified life and a nurturing relationship to the realities and knowledge of diverse groups within a non-racist and non-patriarchal framework.

### 7.3. Challenging imperialism

Recognizing the impact that imperialism has had on the Vietnamese diaspora, we must grieve both individually and collectively. This involves healing from multigenerational trauma and intergenerational conflict within the family and community because of political differences, differences in life experiences, and differences among waves of refugees. Personally, this requires addressing the tension between my parents lived experiences surviving the war and my experiences growing up in Canada. A rupture exists between generations in terms of understanding one another. I feel guilty for not having gone through what my family did and sadness for the sacrifices they made. There is a lack of a greater discussion of the war in Vietnam, the global contributors of the war, and what led to hundreds of thousands of people fleeing their homes, which erases these realities.

In their discussion of Vietnamese diasporic families, Ly (2019) states that refuge is both materially and discursively geographic by how it promises safety and freedom to exist within the humanist cartography of family and the constructed family of the nation-state. She writes about the ways in which exile and displacement are forms of solitude. Specifically, refugees are distinct from other exiled migrants as their institutionalization as a legal category constitutes recognizable persecution and a violation of human rights (Ly, 2019). Similar to other migrants from nations in the Global South, it is generally understood that refugees are forcibly separated from the family of the nation-state and assumed to desire a sense of belonging in those institutions. As a result of how settler colonial states such as the US and Canada have put parameters on the legal movement into their borders through family reunification policies and private sponsorship from groups such as churches, migrants are made vulnerable to heteronormative and patriarchal regulatory structures (Ly, 2019).

Following the breakdown of the welfare state and the increase in neoliberal policies like the Immigration Act of 1990, as Reddy (2005) suggests, the family unit is often the predominant way migrant gain support and vital services such as employment, health care, and housing. As such, the concept of refuge supports the naturalization of attachments to the liberal humanistic, white supremacist concept of family (Ly, 2019). Ly (2019) discusses how this concept of family, which is heteronormative and homonormative, acts as a framework for refugee sociality, as family is assumed to be lost or broken as a result of displacement and due to the ways in which the state constructs the family as a prerequisite for settlement. The silence and tension within our families is in conflict with the idea of family as a space of safety and belonging.

### 7.4. Challenging racialization

To subvert racialization and racial hierarchies, the government needs to recognize Vietnamese people as leaders, acknowledging our contributions to our communities and to Canada. As a collective, we must find ways to resist acculturation and assimilationist policies and practices to preserve our culture and traditions. We must create relationships across racial lines to resist racial and ethnic discrimination from Canadians. In addition, there is a need to resist the internalization and reproduction of the racist structures of the Canadian nation state through participation in racist acts even while experiencing discrimination ourselves as members of the Vietnamese diaspora. Engaging in the promotion of our cultural identity and knowledge is crucial to our survival. I seek to resist the model minority stereotype, and, in my classroom, I teach about racism (personal, cultural, systemic/structural) and how it plays out in our everyday life. This involves defining, negotiating, and claiming our own identities while recognizing the multiplicities and complexities of identities and relations within the community, which gets flattened in a multicultural society (Ngo, 2019a). In the Canadian context, solidarity building with Indigenous and other racialized groups while honoring our differences is so important.

## 8. Conclusion

Research (e.g., Ngo, 2016a) and my own experiences of community work with local Vietnamese Canadians suggests that we are burdened by trauma, distrust, and war-created divisions. This plays out through familial and community silences and mistrust. My experiences have shown me how important it to address the intersectional issues of resisting imperialism and colonialism, economic justice, accountability, and healing from racialized trauma to create a holistic vision of the conditions that support healthy, equitable, and loving relationships. This involves resistance to ongoing marginalization by naming and addressing systemic discrimination and colonial violence, while healing and cultivating collective care through storytelling, organizing and solidarity, connection, and mutual aid. Resisting ongoing marginalization then becomes a collective responsibility rather than an individual, and the possibilities for deepening relationship and accountability with and for one another in the face of oppression grows.

The last few years of a global pandemic have demonstrated how interconnected our world is and how the legacies of colonialism, war, and economic exploitation continue to reproduce the status quo and disparities in nations in the Global South and even within the borders of the high-income nations in the Global North. This has resulted in the mass migration of refugees and immigrants, including Syrian refugees whose experiences are remarkably similar to those of Vietnamese refugees. Both waves of forced displacement were driven by people who felt unsafe in their home country due to war and escaped violence by fleeing by boat. Future research should focus on the parallels and intersections between the experiences of Vietnamese refugees and contemporary refugees to inform policy and practice. Understanding our lived experiences does not begin with our arrival in Canada, but with our histories from Vietnam. In this way we center the knowledge of Vietnamese people as important sources of information. A major strength of the Vietnamese community are the transnational and international connections to the Vietnamese diaspora across the globe. My family have relationships that extend beyond borders and the boundaries they produce. They are in contact with family and acquaintances who are a part of the diaspora in Canada, the US, and Australia as well as people who live in Vietnam. These relationships transcend geographical and national boundaries. They facilitate the preservation of our culture and identity.

At the same time, they are sites of struggle and rupture. As someone who was born in Canada, I am also a member of a South Vietnamese refugee family of Chinese ethnicity. We have our histories of war, fleeing, and challenging times resettling. This has resulted in internal family conflict and a sense of isolation from others from the greater Vietnamese community. What exacerbates these issues is silence among family members and community members. The things that are left unsaid result in assumptions and feelings of shame, anger, and resentment, which we bury down because we do not feel as though they are important or that our experiences matter (Hong, 2020).

There is a delicate balance of preserving our culture and identity and adapting to Western society to prove ourselves. I have learned to codeswitch between my community and academic/professional spaces. I do not feel like I belong anywhere. As I try to figure out my life, my journey, I work to create a space for myself where I can honor my family's lived experiences while honoring my own unique experience as a second-generation Chinese-Vietnamese person. Part of this includes figuring out what this all means to me and how it affects my family. On the one had I am a part of the collective, but on the other hand I have privileges that my parents never dreamed of when they were on the run. Currently, the media and government have pushed forth concerns about terrorism and national security, and this has led to the problematization of refugees and perception of them as threats. Stroking these state produced fears can allow for extraordinary measures to prevent refugees from settling in Canada, which are supported by the public. As we think of how to support people who have fled their homes or left because they wanted to, we must move away from white supremacist, patriarchal systems of surveillance and the erasure of our experiences. A better route would be to support the development of informal networks of care, following the lead of the community and providing resources while eliminating systemic barriers which prevent them from becoming citizens or having access to education and employment.

## Data availability statement

The original contributions presented in the study are included in the article/supplementary material, further inquiries can be directed to the author.

## Ethics statement

Ethical approval was not required for the study in accordance with the local legislation and institutional requirements. Written informed consent to participate in this study was not required in accordance with the national legislation and the institutional requirements.

## Author contributions

JM conceptualized and designed the study, conducted the critical autoethnography, interpreted the data, and drafted and revised the manuscript.
